# Supplementary material for: Piezo Voltage Controlled Planar Hall Effect Devices
Source: Sci Rep. 2016 Jun 22;6:28458. doi: 10.1038/srep28458 (PMC4916466; doi:10.1038/srep28458)
Supplement: Supplementary Information [file srep28458-s1.doc]

Supplementary Information

Piezo Voltage Controlled Planar Hall Effect Devices

Bao Zhang^1^, Kang-Kang Meng^2^, Mei-Yin Yang^1^, K. W. Edmonds^3^, Hao Zhang^1^, Kai-Ming Cai^1^, Yu Sheng^1,4^, Nan Zhang^1^, Yang Ji^1^, Jian-Hua Zhao^1^, Hou-Zhi Zheng^1^, Kai-You Wang^1*^

*1 SKLSM, Institute of Semiconductors, CAS, P. O. Box 912, Beijing 100083, People’s Republic of China*.

*2 School of Materials Science and Engineering, University of Science and Technology Beijing, Beijing 100048, China*

*3 School of Physics and Astronomy, University of Nottingham, Nottingham NG7 2RD, United Kingdom*.

*4 Department of Physics, School of Sciences, University of Science & Technology Beijing, Beijing 100048, China*.

* Correspondence and requests for materials should be addressed to K. W. (e-mail: kywang@semi.ac.cn).

**Table of Contents:**

**S1. Piezo voltages induced uniaxial deformation of PZT.**

**S2. The magnetic hysteresis loops under different piezo voltages.**

**S3. The magnetocrystalline anisotropy constants *K_C_*, *K_U_* and *K_P_*.**

**S4. The in-situ measured output Hall voltages of the NOR gate.**

**S5. The response time of the piezo voltage controlled device**

**S1. Piezo voltages induced uniaxial deformation of PZT**

The substrate GaAs of the Co_2_FeAl devices were first polished down to 100 ± 10 μm to ensure the deformation can be transferred to the device. Then the Co_2_FeAl devices were bonded to the piezoelectric ceramic transducer (PZT) using two-component epoxy glue. The induced deformation along [110] orientation was measured by strain gauge, which is shown in Supplementary Fig. S1. The positive/negative piezo voltages were found to produce a uniaxial tensile/compressive strain in the direction of [110], which is linearly changed with the applied voltage with the deformation of 1.2×10^-5^ V^-1^.


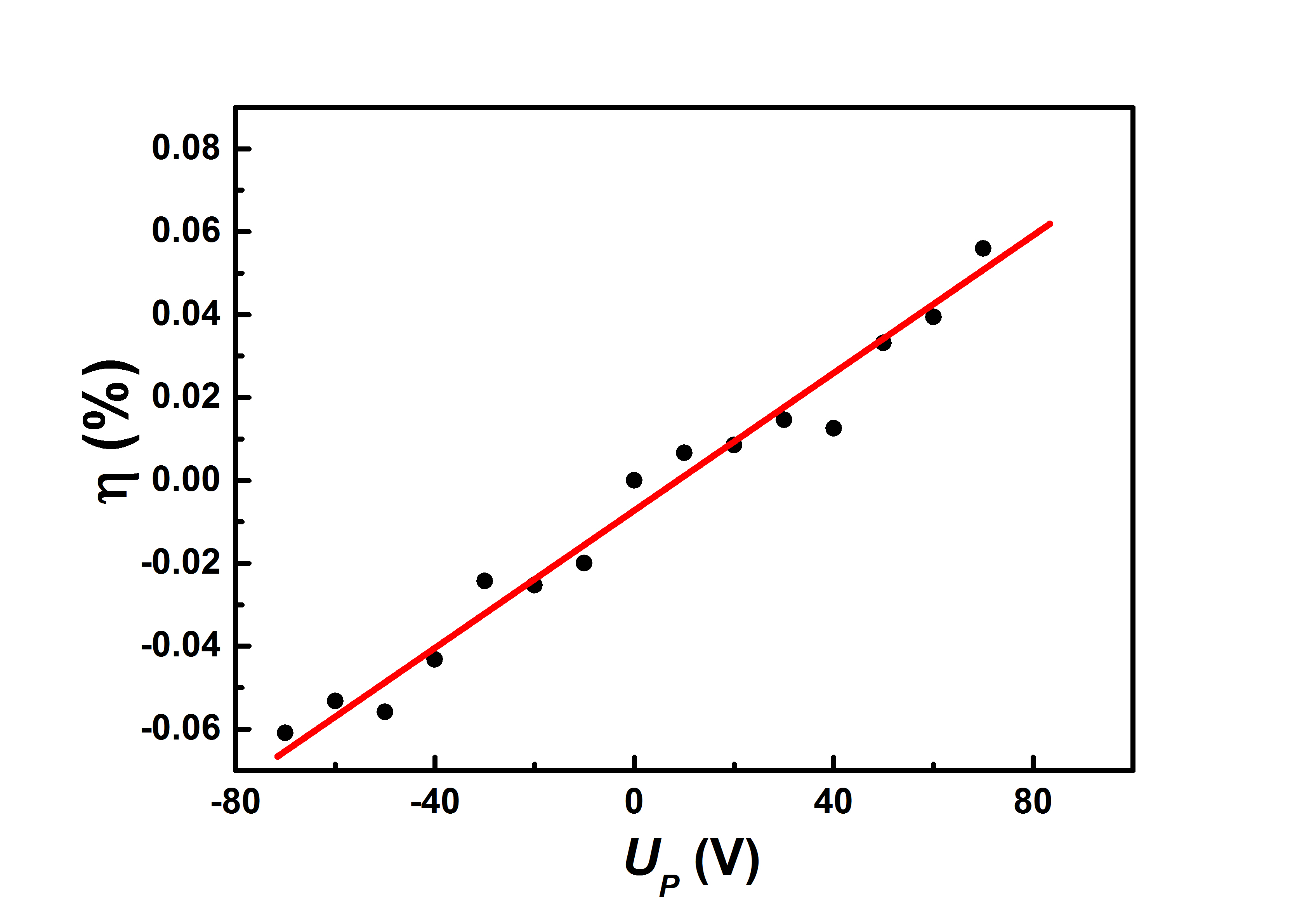


**Supplementary Figure S1. The linear deformation of the Co_2_FeAl devices bonded on the PZT was measured by strain gauge at different voltages.** The device is under stretched / compressed at positive/negative piezo voltages with the deformation factor of $1.20\times10^{-5}V^{-1}$.

**S2. The magnetic hysteresis loops under different piezo voltages**

In order to know how the magnetic anisotropy change of the devices under piezo voltages more clearly, the magnetic hysteresis loops along in-plane [110] and $[\bar{1}10]$ orientations under different piezo voltages have been compared with respect to the virgin state. The magnetic hysteresis loops were performed by using longitudinal magneto-optical Kerr microscopy. With the magnetic field applied in [$\bar{1}10$] orientation at different piezo voltages (*U_P_* = 40, 30, 0, -10, -30 V) as shown in Supplementary Fig. S2a, the two step jumping were observed in the hysteresis curves except for the one at *U_P_* = -30 V. However, the continuous reversible magnetic field range between the two sharp jumps monotonously decreases with decreasing the piezo voltages from *U_P_* = 40 V to lower values. Also the continuous reversible magnetic field range between the two-step jumps shrinks with decreasing the applied the piezo voltages the two-step behavior. Interestingly, the one step switching behavior was observed for *U_P_* = -30 V, indicating the magnetic easy axis has been converted into [$\bar{1}10$] orientation from [110] orientation by the piezo voltage.

When magnetic field was applied in [110] orientations as shown in Supplementary Fig. S2b, the square sharp magnetization reversal was observed when the piezo voltages at *U_P_* = 0, 30, and 40 V, while the coercive field is strongly enlarged at *U_P_* = 40, 30 V, indicating that the magnetic uniaxial of has been enhanced by positive piezo voltages. The axes is changed to harder as shown the cyan and blue loops in the Supplementary Fig. S2a. With the piezo voltage at -30 V, the magnetic hysteresis loop was converted into two-step jump during the magnetization reversal, indicating the magnetic easy axis has been switched by 90^0^ from [110] orientation to orientation by -30 V piezo voltage.


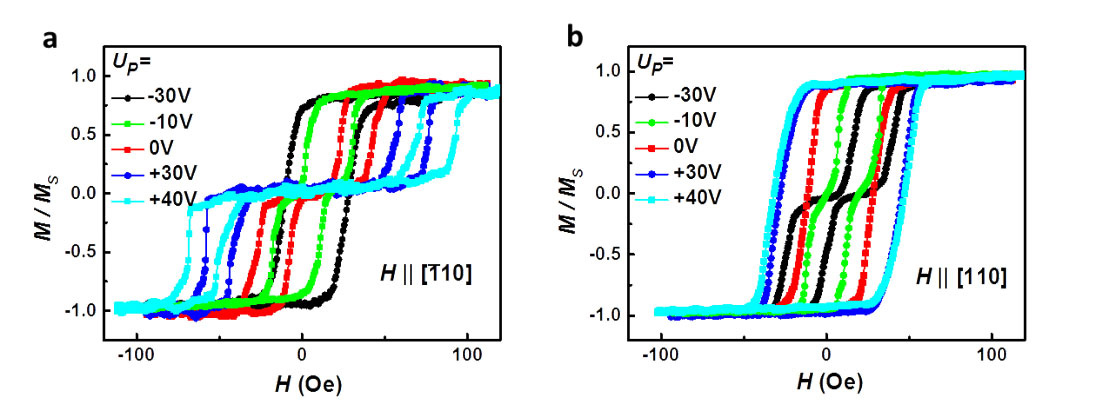


**Supplementary Figure S2. The hysteresis loops with magnetic field along both and [110] orientations under different piezo voltages**. **(a)** The hysteresis loops measured using LMOKM with magnetic field applied in orientation with *U_P_* at 40 (cyan), 30(blue), 0 (red), -15(green) and -30 V (black). **(b)** The hysteresis loops measured using LMOKM with magnetic field applied in [110] orientation with *U_P_* at 40 (cyan), 30(blue), 0 (red), -15(green) and -30 V (black).

**S3. The magnetocrystalline anisotropy constants *K_C_*, *K_U_* and *K_P_***

The magnetic energy of the Co_2_FeAl device without piezo voltages can be described in a phenomenological model that includes a biaxial anisotropy, a uniaxial anisotropy, and a Zeeman term in the energy:

(1)

where *θ* is the angle between magnetization and [110] direction, α is the angle between the external magnetic field and [110] direction, *K_C_* is cubic anisotropy, *K_U_* is the uniaxial anisotropy, *M*_S_ is the saturated magnetization. When the piezo voltage is 0V, the easy axis is along the [110] and the hard axis is along the [010]. The magnetic anisotropic constants can be obtained through the slope of the two-step loop with magnetic field applied in the uniaxial hard orientation (in the Supplementary Fig. S3). The *K_C_* and *K_U_* can be described as follows:

(2)

(3)

where *H_S_* is so-called split field and s is the constant slope between *H_S_* and *-H_S_*. The H_S_ is the start point of the loop where the magnetization vector continuous rotates close to the magnetic uniaxial easy axis when the magnetic field decreasing from the positive to negative and the *-H_S_* is the start point of the loop with magnetization continuous rotation around the magnetic uniaxial easy axis when the magnetic field increasing from the negative to positive. The split field *H_S_* and the slope *s* can be directly obtained from the experimental hysteresis loop, then the *K_C_* and *K_U_* can be obtained using equation (3 and 4) to be (108±5)*M_S_* and (41±2)*M_S_*, respectively. With a piezo voltage applied to the PZT, an extra uniaxial anisotropy will be introduced into the magnetic energy, which can be written down as follow:

$\varepsilon(\theta)=\frac{1}{4}K_{C}\sin^{2}(2\theta)+K_{U}\sin^{2}(\theta)+K_{P}\sin^{2}(\theta-\gamma)-HM_{s}\cos(\theta-\alpha)$ (4)

With piezo voltages applied, the extra uniaxial anisotropy *K_P_* has the same sign as the intrinsic *K_U_* at positive piezo voltages, while *K_P_* has the opposite sign to *K_U_* at negative piezo voltages. Because the induced uniaxial anisotropy has similar format to that of the *K_U_*, we thus can still use equation (2) and (3) to calculate the magnetic anisotropy. Using equation (4), the biaxial anisotropy is independent to the piezo voltages, which were obtained to be (110±5)*M_S_* and (110±5)*M_S_* with piezo voltages at +30 and -30 V, respectively. It is worth noting that the *K_U_* in equation (3) should be *K_U_+K_P_* with piezo voltage at +30 V. The obtained *K_P_* is (26±1)*M_S_* with piezo voltage at +30 V. However, the magnetic easy axis has been converted into orientation with applied piezo voltage at -30 V. The *K_U_* in equation (3) is still valid with *K_U_* = |*K_U_+K_P_*|, where *K_P_* is larger than *K_U_* and they have opposite sign. Thus the obtained *K_P_* is (-46±4)*M_S_* with piezo voltage at -30V. In a word, the piezo voltage induced extra uniaxial anisotropy can effectively tune the magnetic easy axis through the magnetic energy of the system.


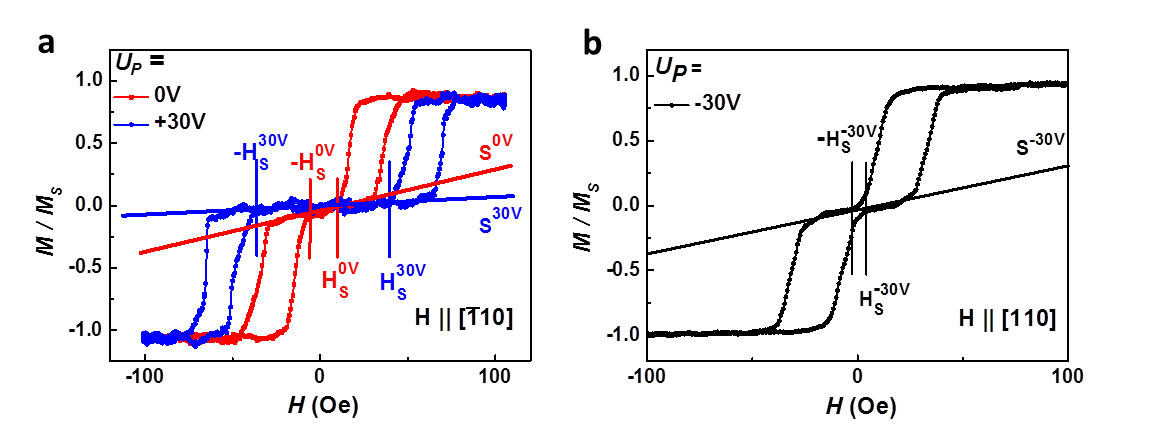


**Supplementary** **Figure S3. The magnetic hysteresis loops for Co_2_FeAl device under piezo voltages. (a)** The magnetic hysteresis loop of Co_2_FeAl device with magnetic field applied along direction with *U_P_* at 0 (red dots) and +30V (blue dots).The split field and slope *H_s_* and *s* with *U_P_* at 0 V (marked with red curves) and +30 V (marked with blue curve), respectively. **(b)** The magnetic hysteresis loop of Co_2_FeAl film with magnetic field applied along [110] direction with *U_P_* at -30 V (black dots), the split field and slop are *H_S_* and s (marked with dark curves), respectively.

**S4. The in-situ measured output Hall voltages of the NOR gate**

The NOR gate was built based on one [100] and one [010] planar Hall effect device connected as shown in Figure 3c in the main text. The two piezo voltages (*U_P1_* and *U_P2_*) control the [010] and [100] orientated devices separately. The magnetizations of two devices were preset to [110] orientation by external magnetic field. Then all the operations were executed without external magnetic fields. In this experiment, the output planar Hall voltages of the NOR gate at different piezo voltage configurations are shown in figure S4, where four state values are observed under different piezo voltages. The non-zero value of Hall voltage with *U_P1_* = -30V, *U_P2_* = 0V and *U_P1_* = 0V, *U_P2_ =* -30V is caused by the slightly different of two Hall devices.


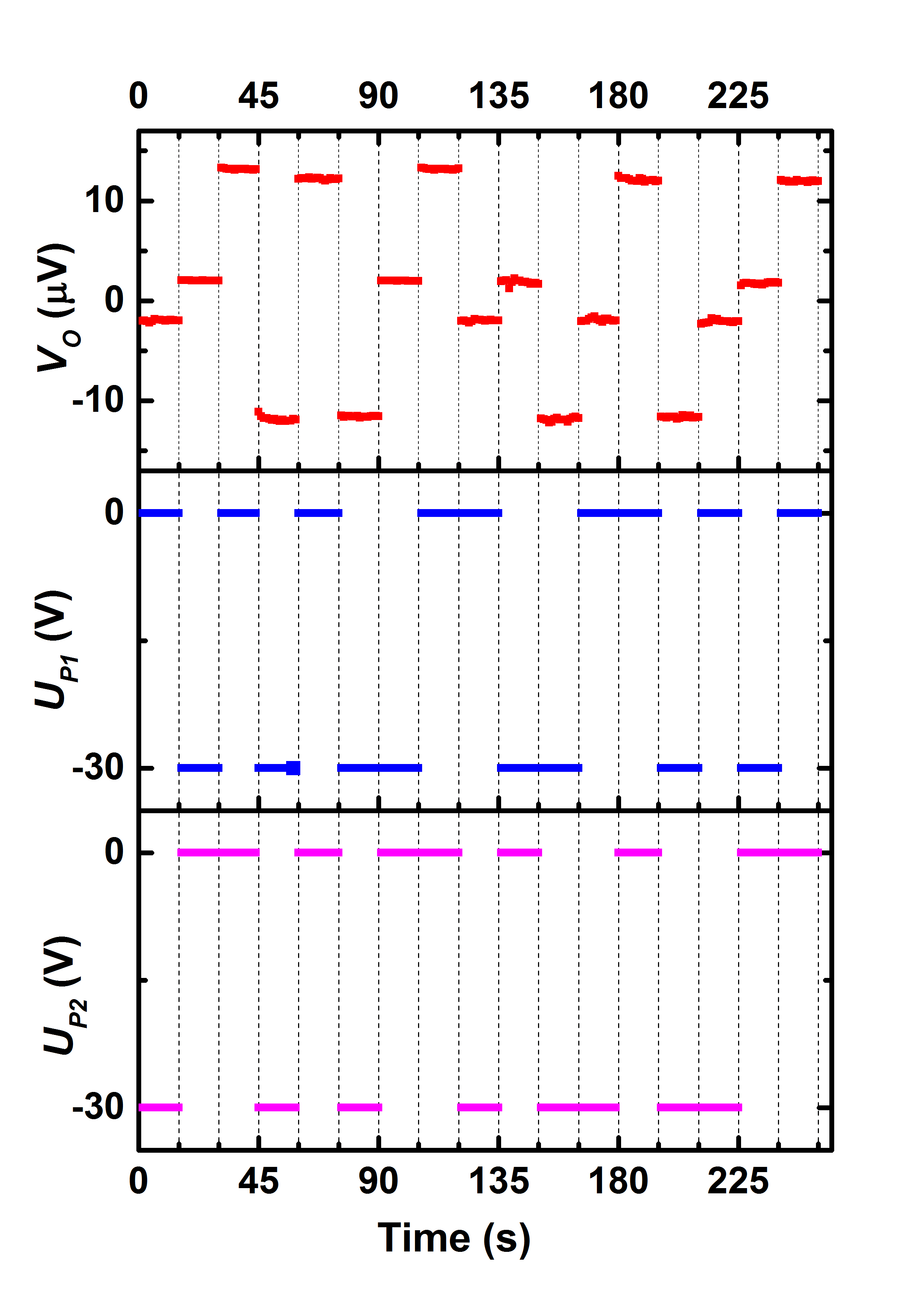


**Supplementary Figure S4. Programmable logic operation demonstrated by a NOR gate.** The output voltages of the NOR gates are described depending on the measured values with varying the piezo voltages *U_P1_* and *U_P2_*, where four states are observed.

**S5. The response time of the piezo voltage controlled device.**

Using time dependent Kerr signal under piezo voltage pulse control, we investigated the time-dependent response of piezo voltage controlled planar Hall devices. The response Kerr signals with rising (with piezo voltage change from 0 to -30 V) and falling (with piezo voltage change from -30 to 0 V) piezo voltage pulses for [110] oriented device is shown in Fig. S5. The rising and falling dynamics can be expressed by$M(t)=M_{0}[1-\exp(-t/\tau_{r})]$ and$M(t)=M_{0}\exp(-t/\tau_{d})$, respectively, where $\tau_{r}$and $\tau_{d}$are the time constants for the rising and falling responses, respectively, $M$is the Kerr rotation and $M_{0}$is a constant for the maximum Kerr rotation angle of 90^0^ rotation of the magnetization. The rising and falling time of the piezo voltage controlled device are 220 μs and 70 μs, respectively.


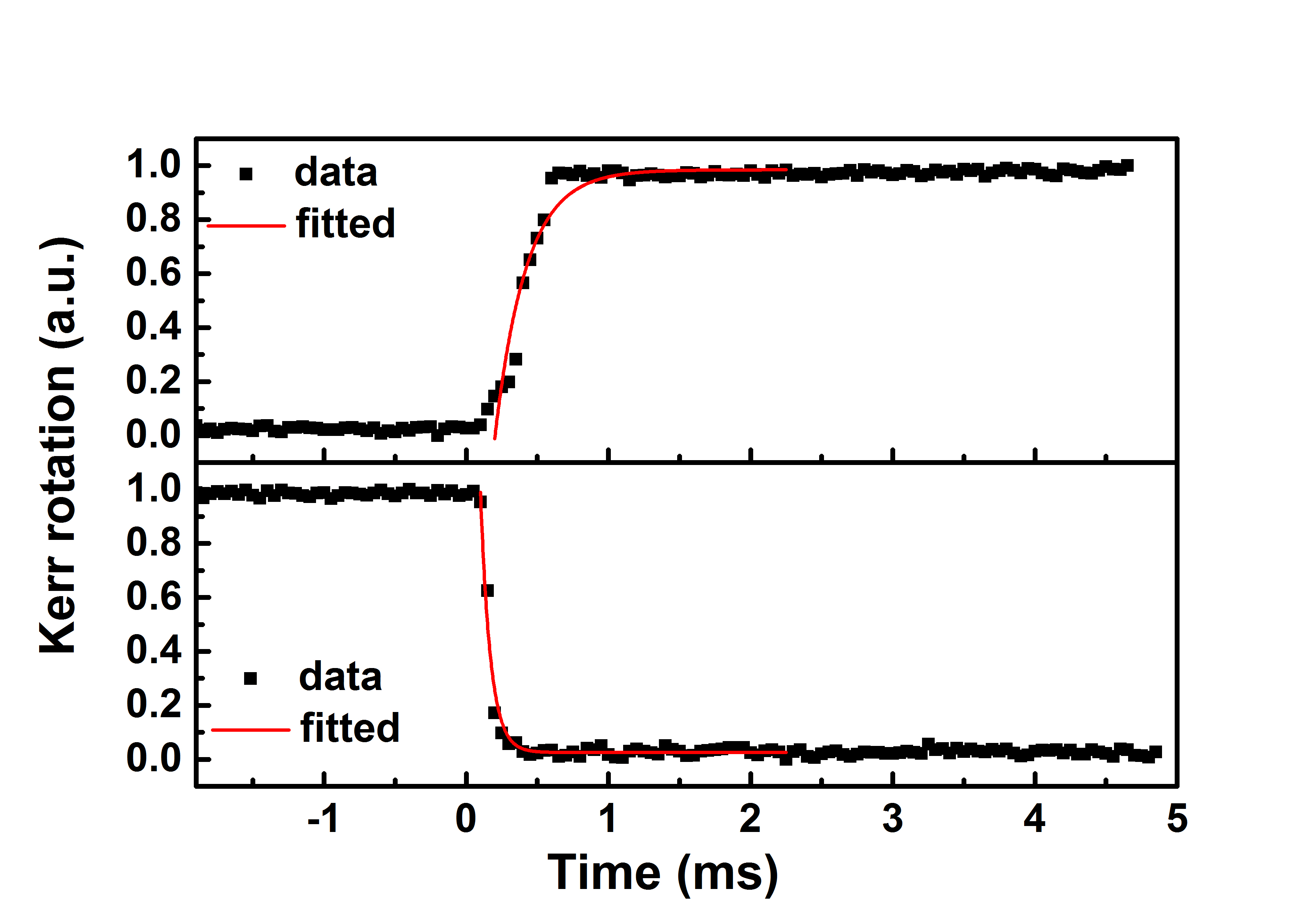


**Figure S5. The response time of the piezo voltage controlled device.** The solid dots are the experimental results, and the lines are the fitting curves. The rising and falling time are 220 μs and 70 μs, respectively.
